# Improved Kaempferol Solubility via Heptakis-O-(2-hydroxypropyl)-β-cyclodextrin Complexation: A Combined Spectroscopic and Theoretical Study

**DOI:** 10.3390/ijms252312492

**Published:** 2024-11-21

**Authors:** Dongxu Han, Zhongbao Han, Liyan Liu, Shigang Xin, Zhan Yu

**Affiliations:** 1 School of Chemistry and Chemical Engineering, Shenyang Normal University, Shenyang 110034, China; hdx17824030075@163.com (D.H.); yuzhan@synu.edu.cn (Z.Y.); 2 Experimental Teaching Center, Shenyang Normal University, Shenyang 110034, China; xsg3948@126.com

**Keywords:** kaempferol, HP-β-CD, phase solubility, molecular dynamics, PM7

## Abstract

Four cyclodextrins (CDs) including heptakis-O-(2-hydroxypropyl)-β-cyclodextrin (HP-β-CD), heptakis-O-(2,6-di-O-methyl)-β-cyclodextrin (DM-β-CD), β-cyclodextrin (β-CD), and γ-cyclodextrin (γ-CD) were evaluated for their ability to enhance the aqueous solubility of kaempferol (Kae). Phase solubility studies indicated that these four CDs can form 1:1 type complexes with Kae and that HP-β-CD demonstrated the most significant solubilizing effect on Kae. Among the CDs tested, HP-β-CD demonstrated the most significant solubilizing effect on Kae. With an HP-β-CD concentration of 5.00 × 10^−3^ mol·L^−1^, the concentration of Kae reached 4.56 × 10^−5^ mol·L^−1^, which is 12.7 times greater than its solubility in water. Characterization of the HP-β-CD/Kae complex was performed using empirical methods. Molecular docking indicated that the A and C rings of Kae fit into the hydrophobic cavity of HP-β-CD, while the B ring remained at the rim. Six hydrogen bonds were found between HP-β-CD and the -OH groups of Kae. The negative complexation energy (Δ*E*) suggests the complex formation was exergonic. A 30-ns molecular dynamics simulation revealed no significant structural changes, with average root-mean-square deviation RMSD values of 2.230 Å for HP-β-CD and 0.786 Å for Kae, indicating high stability of the complex.

## 1. Introduction

**Figure 1:**
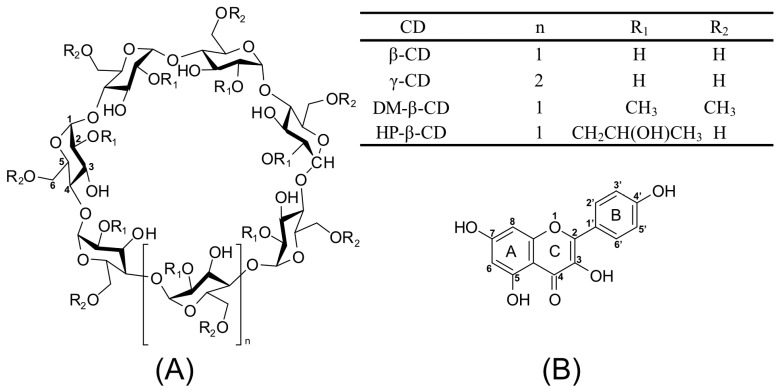
Chemical structures of CDs, including β-CD, γ-CD, DM-β-CD and HP-β-CD (**A**) as well as Kae (**B**).

Cyclodextrins (CDs, shown in Figure 1A) [1,2] are natural cyclic oligosaccharides, classified into three primary categories based on the number of D-(+)-glucopyranose units linked by α-1,4 glycosidic bonds: α-cyclodextrin (α-CD), β-cyclodextrin (β-CD), and γ-cyclodextrin (γ-CD). Specifically, α-CD comprises six units, β-CD seven units, and γ-CD eight units [3]. CDs deviate from a perfect cylindrical form because the chemical bonds between glucopyranose units lack free rotation; instead, they exhibit a toroidal or cone-like configuration [4]. They display a hydrophilic nature with hydroxyl groups on their external surface and a hydrophobic cavity at the center. This structural uniqueness allows CDs to form non-covalent inclusion complexes by encapsulating guest molecules partially or fully within their central cavity, which can greatly affect the solubility, stability, and physicochemical characteristics of the guest [5]. However, the inherent hydrogen bonding within natural CDs results in poor water solubility, a property that presents a significant challenge to their potential applications in various fields. To address this limitation, chemically modified CDs such as heptakis-O-(2-hydroxypropyl)-β-cyclodextrin (HP-β-CD) and heptakis-O-(2,6-di-O-methyl)-β-cyclodextrin (DM-β-CD) have been developed. These derivatives, along with their inclusion complexes, are widely applied in fields like food, pharmaceuticals, and cosmetics [6].

Kaempferol (Kae, shown in Figure 1B), also known as hawthorn flavonol, is primarily found in the roots of hawthorn [7]. It has limited solubility in water and can be found in a variety of fruits and vegetables such as strawberries, potatoes, pumpkins, tomatoes, and green beans, among others [8,9]. Additionally, it is abundant in rosemary, ginkgo biloba, forsythia, and other herbs. Studies have demonstrated its effectiveness in inhibiting cancer cell growth [10,11,12]. Kae has anti-inflammatory and antibacterial properties and has been extensively studied for its potential use in treating pathogenic bacterial infections. It has been identified as a promising antiviral candidate for the treatment of Streptococcus pneumoniae co-infections, including those caused by the 2019 novel coronavirus (COVID-19) [13]. Furthermore, it has been observed to affect the survival and apoptosis of osteoblasts and to inhibit bone loss. However, Kae has low solubility in aqueous media [11], which reduces its bioavailability. As a result, Kae is typically administered orally as an aqueous suspension, limiting its clinical applications [14,15].

The aim of this research is to explore the improvement of Kae’s solubility in water by forming a complex with cyclodextrins (CDs). Four different CDs—β-CD, γ-CD, HP-β-CD, and DM-β-CD were utilized as solubilizers. The results revealed that HP-β-CD proved to be the most effective in enhancing Kae’s solubility. To analyze the HP-β-CD/Kae inclusion complex, techniques such as UV-Vis spectroscopy, Fourier transform infrared spectroscopy (FT-IR), X-ray diffraction (XRD), and thermogravimetric analysis (TGA) were employed. Molecular docking was used to determine the potential structural configuration of the HP-β-CD/Kae complex. Additionally, a 30-ns molecular dynamics simulation was carried out on the lowest energy docking result to evaluate the complex’s stability.

## 2. Results and Discussions

### 2.1. Phase Solubility Study

**Figure 2:**
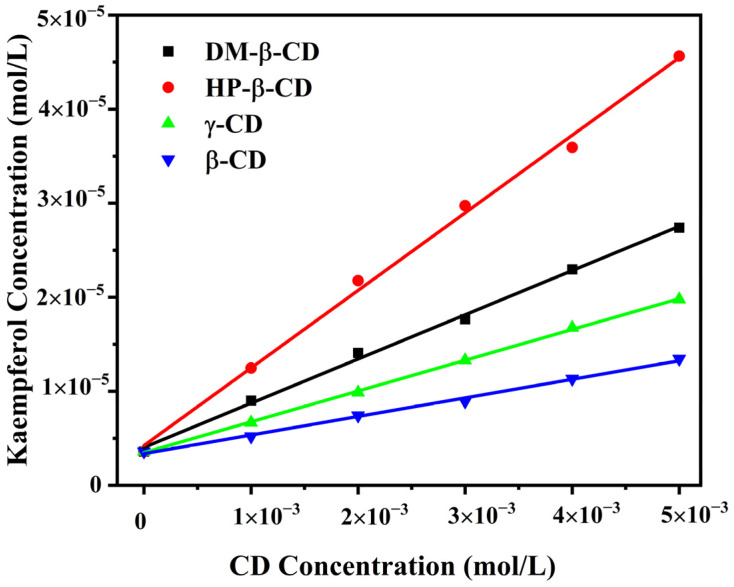
Phase solubility diagrams of Kae in aqueous solution with the presence of various CDs.

A phase solubility diagram was generated by recording the molar concentration of Kae in the presence of four different CDs across varying concentrations, as illustrated in Figure 2. Kae’s solubility exhibited a linear increase as CD concentrations rose, with concentrations of CD ranging from 0.0 to 5.0 × 10^−3^ mol·L^−1^. This increase in solubility confirms the presence of intermolecular interactions between the host (CD) and the guest (Kae). Additionally, all the solubility profiles correspond to the A_L_ type, based on the classification system by Higuchi and Connors [16], signifying the formation of 1:1 molecular complexes.

The apparent stability constant (*K*) of the host-guest complex serves as an indicator of the binding strength between the host and guest molecules. The equilibrium constants (*K* values) for the complexes were derived from the linear phase solubility plots and are shown in Table 1. Among the four complexes, the HP-β-CD/Kae complex had a *K* value of 2311.69 L/mol, which is higher than the value reported by Mercader et al. [17] using a suspension method. The solubility of Kae increased by a factor of 12.7 when HP-β-CD was used at a concentration of 5.00 × 10^−3^ mol·L^−1^.

### 2.2. Fourier Transform Infrared Spectroscopy

**Figure 3:**
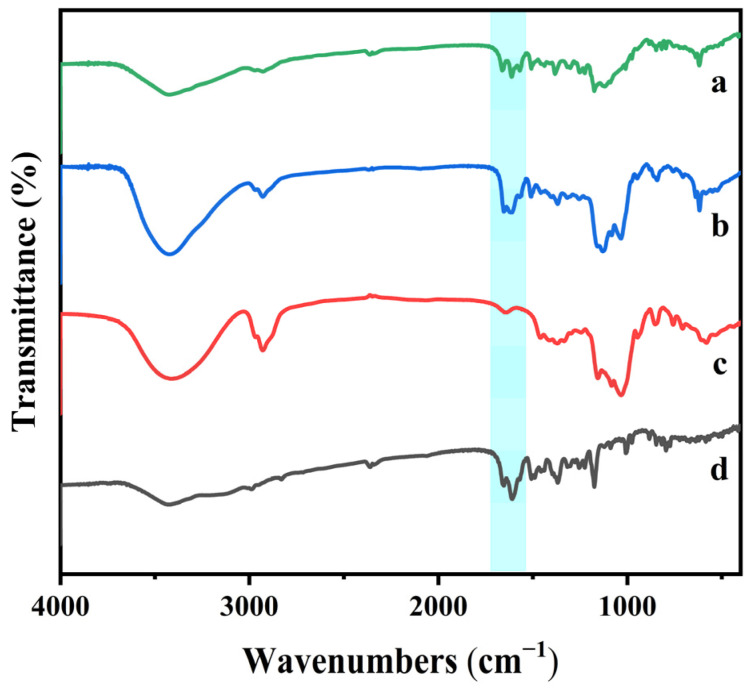
FT-IR spectra of HP-β-CD/Kae inclusion complex (a), HP-β-CD/Kae physical mixture (b), HP-β-CD (c), and Kae (d).

Figure 3 displays the FT-IR spectra of four samples: the HP-β-CD/Kae complex, the HP-β-CD/Kae physical mixture, HP-β-CD, and Kae. The FT-IR spectrum of HP-β-CD showed major absorption bands at 3415 cm^−1^ (O-H stretching), 2930 cm^−1^ (C-H stretching), 1649 cm^−1^ (H-O-H bending), 1160 cm^−1^ (C-O stretching), and 1031 cm^−1^ (C-O-C stretching). These observations are consistent with those reported by Liu and colleagues [6]. Kae’s FT-IR spectrum exhibited key bands at 1658 cm^−1^ (C-C stretching of the A ring), 1616 cm^−1^ (C=O stretching), 1510 cm^−1^ (C=C stretching of the A ring), 1373 cm^−1^ (C-C stretching), 1178 cm^−1^ (C-C stretching of the B ring), and 1010 cm^−1^ (C-O stretching of the B ring), aligning closely with previously reported spectra [18].

The physical mixture of HP-β-CD and Kae displayed a spectrum that exhibited the typical characteristic bands of both compounds (3420, 2929, 1662, 1594, 1521, 1380, 1328, and 1030 cm^−1^). These findings indicate minimal interaction between the two compounds in the physical mixture, suggesting that HP-β-CD and Kae do not interact significantly in this form. In contrast, the inclusion complex of HP-β-CD and Kae showed notable changes in the absorption bands, with those of Kae at 1658, 1616, and 1510 cm^−1^ shifting to 1668, 1612, and 1505 cm^−1^, respectively. This suggests that the aromatic A ring, O–H, and C=O groups of Kae may play a crucial role in mediating the interaction between HP-β-CD and Kae.

### 2.3. X-Ray Diffraction (XRD)

**Figure 4:**
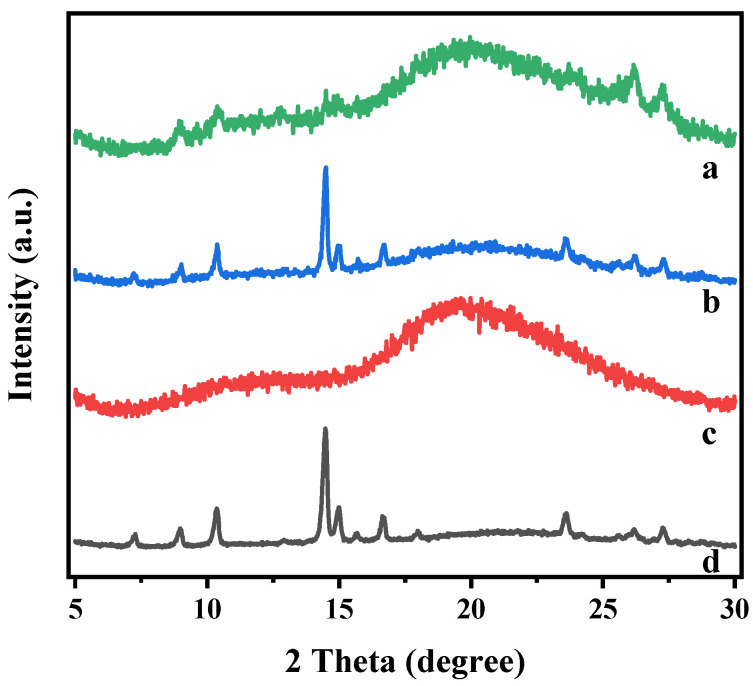
XRD patterns of HP-β-CD/Kae inclusion complex (a), HP-β-CD/Kae physical mixture (b), HP-β-CD (c), and Kae (d).

Figure 4 illustrates the X-ray diffraction (XRD) patterns of Kae, hydroxypropyl-β-cyclodextrin (HP-β-CD), their physical mixture, and an inclusion complex. Kae exhibited sharp peaks at 7.26, 8.96, 10.32, 14.50, 15.00, 15.62, 16.66, 18.00, 23.56, 26.22, and 27.28°, indicating the crystalline nature of this compound. HP-β-CD displayed a broad peak at 19.50°, suggests its amorphous nature. The physical mixture’s XRD pattern combined features of both components, confirming no chemical interaction and that both retained their original physical properties. In contrast, the HP-β-CD/Kae complex showed an amorphous halo pattern, indicating complete encapsulation of Kae within the HP-β-CD cavity [19,20].

### 2.4. Thermogravimetric Analysis

**Figure 5:**
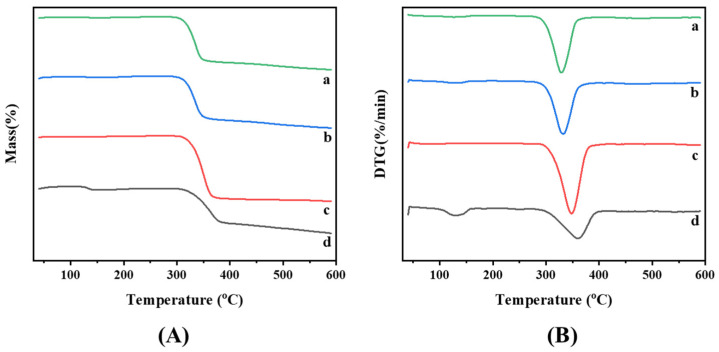
TG (**A**) and DTG (**B**) curves of HP-β-CD/Kae inclusion complex (a), HP-β-CD/Kae physical mixture (b), HP-β-CD (c), and Kae (d).

Figure 5 shows the TG and DTG curves for the HP-β-CD/Kae inclusion complex, the physical mixture, HP-β-CD, and Kae. Kae showed significant degradation, with mass losses of 48.46% between 353–420 °C and 13.70% between 400–590 °C, reaching a peak mass variation at 352.63 °C, consistent with previous studies [21]. HP-β-CD displayed minimal mass loss between 25 °C and 300 °C, followed by an 86.86% reduction in the 300–400 °C range, with maximum degradation at 347.6 °C, and a further 3.44% mass loss from 400–590 °C.

The HP-β-CD/Kae inclusion complex showed a peak mass loss rate at 327.62 °C, lower than that of the physical mixture, which peaked at 332.63 °C. This difference in melting points suggests a reduced thermal stability of the HP-β-CD/Kae complex, likely due to guest amorphization during complex formation [22]. Similar findings have been reported in the inclusion processes of other compounds [23,24].

### 2.5. Molecular Modeling Studies

**Figure 6:**
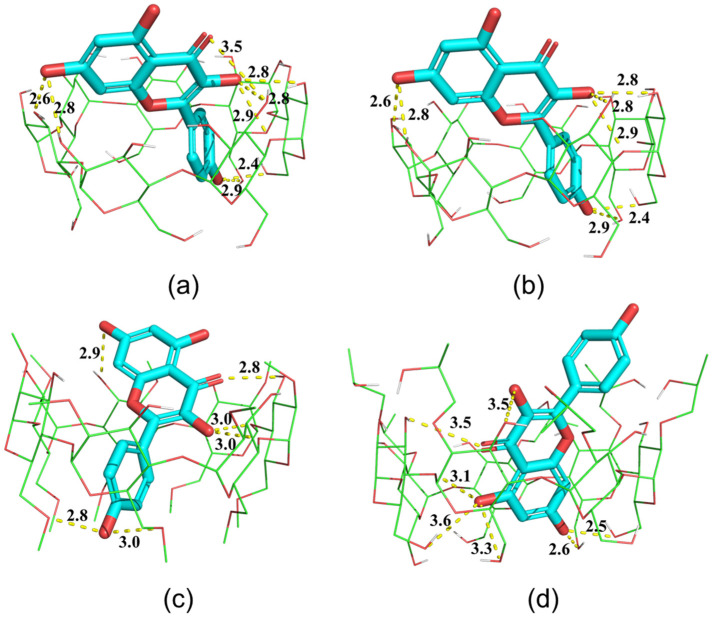
Snapshot of the lowest energy docking result for CD/Kae complexes, where CD is β-CD (**a**), γ-CD, (**b**) DM-β-CD (**c**), or HP-β-CD (**d**). The hydrogen bonds are represented by dashed lines with indicated distances (in Å).

In this study, molecular docking was employed to investigate the formation mechanism of the CD/Kae complex. After performing 300 molecular docking runs, the lowest-energy result yielded the optimal complex structure, as illustrated in Figure 6.

Figure 6b shows that γ-CD, with a cavity 1.6 times larger than that of β-CD [2] and its derivatives, allowed Kae to dock at its secondary rim, with the B ring extending into the γ-CD cavity. In contrast, β-CD and its derivatives provided cavities that were better suited for accommodating Kae. The A and C rings of Kae fit well within the cavities of HP-β-CD, while the B ring was situated within the cavities of β-CD and DM-β-CD. Considering that DM-β-CD has a smaller cavity volume than natural β-CD [25], the docking results appear to be logical.

Figure 6d shows that the A and C rings of Kae were accommodated within the HP-β-CD cavity, while the B ring was positioned at the secondary rim. Numerous hydrogen bonds can be observed between the host and guest, suggesting that both hydrogen bonding and hydrophobic interactions played key roles in forming the HP-β-CD/Kae complex. As also reported by Wang et al. [26], an intramolecular hydrogen bond was observed between the C4 carbonyl group and the C6 hydroxyl group.

The stability of the complex can be assessed by evaluating its complexation energy (Δ*E*) relative to the isolated molecules. In order to achieve this, the binding energy of β-CD/Kae, γ-CD/Kae, DM-β-CD/Kae, and HP-β-CD/Kae complexes was calculated using quantum mechanics at a semi-empirical level with the PM7 methods, the latest parametrization method for MOPAC [27].

Table 2 presents the results of the calculations performed using PM7 methods. In addition to β-CD/Kae, the results obtained from these methods demonstrated a negative Δ*E* on an identical scale, suggesting that the formation energy (*E*) of the inclusion complex is less than the combined formation energies of the isolated host and guest molecules. Among them, HP-β-CD/Kae has the smallest Δ*E* value, indicating that the formation of the complex is thermodynamically most favorable. Nevertheless, the Δ*E* value of β-CD/Kae was the greatest or even greater than zero, indicating that the β-CD/Kae complex was the most challenging to form. This finding aligns with the observation that the solubilizing effect of β-CD on Kae.

**Figure 7:**
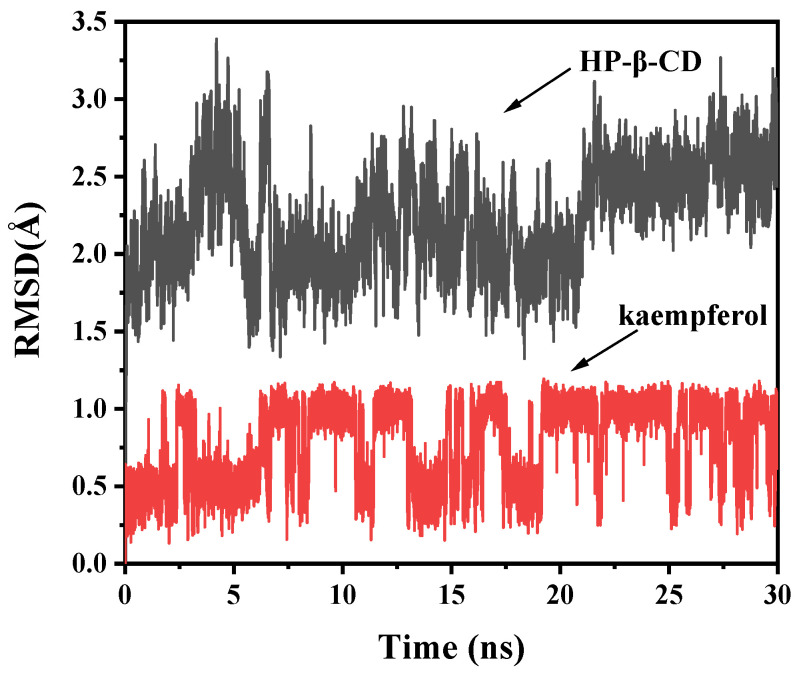
RMSD plots of all atoms for HP-β-CD/Kae complex.

The structural stability of the HP-β-CD/Kae complex obtained from the lowest-energy molecular docking solution was estimated using 30-ns molecular dynamics (MD) simulations. Figure 7 illustrates the average root-mean-square deviation (RMSD) values for both host and guest molecules in relation to their initial positions. The system rapidly stabilized at the start of the MD simulation, with only slight atomic position fluctuations for both the host and guest. The mean RMSD for HP-β-CD and Kae were 2.230(2) Å ± 0.341(8) Å and 0.786(4) Å ± 0.266(0) Å, respectively. These results suggest minimal positional changes and structural deformations for both molecules, confirming the structural stability of the complex as indicated by the lowest-energy molecular docking result.

## 3. Materials and Methods

### 3.1. Materials

Kae was procured from Regal Biological Company (Shanghai, China), while β-CD, γ-CD, HP-β-CD, and DM-β-CD were obtained from Yuanye Biotechnology (Shanghai, China). All other chemicals and solvents used were of analytical grade or higher. Ultrapure water with a resistance of 18.2 MΩ·cm was employed for the preparation of solutions and for the dilution of samples.

### 3.2. Instrumentation

Ultraviolet-visible (UV-Vis) spectra were measured using a UH 5300 UV-Vis spectrometer (Hitachi, Tokyo, Japan) at room temperature, covering a wavelength range of 200–800 nm, with a 1-nm band width and 0.5-nm data intervals.

Infrared spectra were recorded on a Nicolet 380 Fourier transform infrared spectrometer (Thermo Scientific, Waltham, MA, USA) within the 4000–400 cm^−1^ range, with a 2-cm^−1^ resolution and 32 co-added scans.

X-ray diffraction (XRD) data were collected using an Ultima IV X-ray powder diffractometer (Rigaku, Tokyo, Japan) with Cu-Kα radiation (λ = 1.5418 Å), under the following conditions: 40 kV, 40 mA, scanning range 5–30°, and scan rate of 0.02° min^−1^.

Thermogravimetric analysis was conducted using a STA449 F5 thermal analyzer (NETZSCH, Selb, Bavaria, Germany). Samples weighing between 2.0 and 2.5 mg were subjected to a heating process, increasing the temperature from room temperature to 590 °C at a rate of 10 °C per minute.

### 3.3. Phase Solubility Study

A phase solubility study was performed following the method by Higuchi and Connors [16]. An excess of Kae (1.0 mg) was added to centrifuge tubes containing 5.0 mL of distilled water with varying CD concentrations (0–1.0 × 10^−2^ mol·L^−1^). The tubes were sonicated for 30 min to reach equilibrium, then centrifuged at 4000 rpm for 20 min at room temperature. The supernatant was collected and analyzed at 378 nm, with each measurement done in triplicate. The stability constant (*K*) of the solute and solubilizer was determined from the phase solubility diagram using Higuchi and Connors’ equation.

(1) $$K=\frac{slope}{s_{0}(1-slope)}$$

The slope is derived from the plot of Kae concentration versus CD concentration, while s_0_ represents the aqueous solubility of Kae. Based on the results of preliminary experiments, the s_0_ of Kae was determined to be 3.599 × 10^−6^ mol·L^−1^.

### 3.4. Preparation of HP-β-CD/Kae Inclusion Complex and Physical Mixture

The physical mixture of HP-β-CD and Kae was prepared by blending both components in a mortar and pestle until homogeneous [28].

The HP-β-CD/Kae inclusion complex was made using a modified version of the kneading method by Zhang et al. [29]. A small amount of water was added to the HP-β-CD powder in a mortar, and after a few minutes of kneading, a smooth paste was formed. Kae was then gradually mixed into the paste with a minimal amount of ethanol. The mixture was kneaded for over 45 min and then dried at 50 °C for 24 h.

### 3.5. Molecular Modeling, Docking, and Dynamics Studies

Three-dimensional structures of β-CD and γ-CD were retrieved from the Cambridge Crystallographic Data Centre (entries ARUXIU and CYDXPL, respectively). HP-β-CD and DM-β-CD structures were generated from the β-CD structure using PyMol (version number:1.8.6). The 3D structure of Kae was sourced from PubChem (https://pubchem.ncbi.nlm.nih.gov, accessed on 16 August 2024). All structures underwent structural optimization using the MMFF94 force field [30] before molecular simulations. Host-guest interactions were analyzed using Autodock 4.2 [31] from the Scripps Research Institute. Default Autodock settings were applied for rotatable bonds and non-polar hydrogens, and all guest bonds were flexible. The Lamarckian genetic algorithm (LGA) was employed, with key parameters set as: population size of 300, 2,500,000 energy evaluations, and LGA population size of 150.

The lowest-energy conformation of the docking simulation was extracted and subjected to analysis. The MOPAC 2016 program, developed by Stewart Computational Chemistry (Colorado Springs, CO, USA), was used to calculate the complexation energy (Δ*E*). The PM7 force fields were employed [32]. In addition, the MOPAC calculations employed the following supplementary keywords: EF PRECISE CHARGE = 0, GNORM = 0.1, XYZ THERMO (298.15, 298.15), LET. Binding energy (Δ*E*) was defined as the difference between the heat of complex formation and the heat of involved free molecules, as represented by the following equation [33].

(2) $$E=E_{complex}-E_{host}-E_{gust}$$

All molecular dynamics simulations were performed using Desmond 2018.4 (DE Shaw Research, New York, NY, USA) [34]. The lowest-energy conformation from the docking process was placed in a cubic box with a side length of 10 Å, filled with TIP3P water model molecules. The relaxation of the system was achieved by implementing Steepest Descent and the limited-memory Broyden-Fletcher-Goldfarb-Shanno algorithms in a hybrid manner. Production runs were carried out in the NPT ensemble at 300 K and 1.013 bar for 30 ns, without the addition of buffer ions. Temperature and pressure were controlled using a Nose-Hoover thermostat (relaxation time of 1.0 ps) and a Martyna-Tobias-Klein barostat (relaxation time of 2.0 ps). The OPLS3 force field was used throughout the molecular dynamic simulations. The short-range coulombic interactions were analyzed using a cut-off value of 9.0 Å using the short-range method. The final production run was carried out for 30 ns and the trajectory sampling was done at an interval of 1.0 ps.

## 4. Conclusions

This study utilized the phase solubility method to evaluate the solubilization efficiency of β-CD, γ-CD, DM-β-CD, and HP-β-CD for Kae. The results indicated that HP-β-CD exhibited the highest solubilization effect on Kae, forming a 1:1 complex. Furthermore, the HP-β-CD/Kae complex was characterized using FT-IR, XRD, and TG techniques. Molecular docking was employed to explore the potential structure of the complex. The thermodynamics and stability of the complex were assessed through semi-empirical quantum calculations and molecular dynamics simulations based on the most favorable docking outcome. Overall, the findings suggest that HP-β-CD is a potent solubilizing agent for insoluble compounds. Its relatively high solubility in water and low toxicity make it particularly suitable for the development of herbal medicines and healthcare products.

## Figures and Tables

**Table 1:** The linear regression equation and the stability constants for CD: Kae inclusion complexes.

CD	Linear Regression Function	*R* ^2^	*K* (L/mol)
HP-β-CD	Y = 0.00825 c + 4.23806 × 10^−6^	0.9961	2311.69
DM-β-CD	Y = 0.00470 c + 4.03346 × 10^−6^	0.9970	1312.26
β-CD	Y = 0.00198 c + 3.37478 × 10^−6^	0.9951	551.32
γ-CD	Y = 0.00327 c + 3.48283 × 10^−6^	0.9995	911.69

**Table 2:** Free energy (*E*) and binding energy (∆*E*) were calculated for the CD / Kae complex using semi-empirical quantum mechanical PM7 methods.

Host	Guest	*E*_complex_ (kcal/mol)	*E*_host_ (kcal/mol)	*E*_guest_ (kcal/mol)	Δ*E* (kcal/mol)
β-CD	Kae	−1596.02(3)	−1376.91(4)	−234.35(6)	15.25
γ-CD		−1710.50(1)	−1469.10(2)		−7.04
DM-β-CD		−1701.83(8)	−1460.99(2)		−6.49
HP-β-CD		−1938.05(2)	−1694.69(8)		−8.99

## Data Availability

All data generated or analyzed in this study have been included in this manuscript. The datasets used and/or analyzed in this study can be obtained upon request from the corresponding authors.
